# The Roles of Bacterial DNA Double-Strand Break Repair Proteins in Chromosomal DNA Replication

**DOI:** 10.1093/femsre/fuaa009

**Published:** 2020-04-14

**Authors:** Anurag Kumar Sinha, Christophe Possoz, David R F Leach

**Affiliations:** Department of Biology, University of Copenhagen, Ole Maaløes Vej 5, Copenhagen, 2200, Denmark; Evolution and maintenance of circular chromosomes, Genome biology department, Institute for Integrative Biology of the Cell (I2BC), CEA, CNRS, Université Paris-Sud, Université Paris-Saclay, 1 avenue de la Terrasse Building 26, 91198 Gif-sur-Yvette, France; Institute of Cell Biology, School of Biological Sciences, University of Edinburgh, King's Buildings, Edinburgh, EH9 3FF, United Kingdom

**Keywords:** *E. coli*, DNA replication, DNA double-strand break repair, Homologous recombination

## Abstract

It is well established that DNA double-strand break (DSB) repair is required to underpin chromosomal DNA replication. Because DNA replication forks are prone to breakage, faithful DSB repair and correct replication fork restart are critically important. Cells, where the proteins required for DSB repair are absent or altered, display characteristic disturbances to genome replication. In this review, we analyze how bacterial DNA replication is perturbed in DSB repair mutant strains and explore the consequences of these perturbations for bacterial chromosome segregation and cell viability. Importantly, we look at how DNA replication and DSB repair processes are implicated in the striking recent observations of DNA amplification and DNA loss in the chromosome terminus of various mutant *Escherichia coli* strains. We also address the mutant conditions required for the remarkable ability to copy the entire *E. coli* genome, and to maintain cell viability, even in the absence of replication initiation from *oriC*, the unique origin of DNA replication in wild type cells. Furthermore, we discuss the models that have been proposed to explain these phenomena and assess how these models fit with the observed data, provide new insights and enhance our understanding of chromosomal replication and termination in bacteria.

## Teaser

The close relationship between bacterial DNA replication and DNA double-strand break (DSB) repair is reviewed here, paying particular attention to recent research in *Escherichia coli* that has revealed two phenomena: DNA amplification of the chromosome terminus due to aberrant reactions in some DSB repair mutants and DNA loss in the chromosome terminus of DSB repair defective mutants.

## INTRODUCTION

In bacteria with circular chromosomes, DNA replication initiates at a fixed position called the origin (*oriC* in *E. coli*) and proceeds bidirectionally to reach the opposite side of the chromosome, where replication forks meet and replication terminates. In the Gram-negative bacterium *E. coli*, replication termination is constrained by 10 *ter* sites, which are bound by the protein Tus. Tus-bound *ter* sites create replication fork traps that allow forks to pass only in the permissive direction and block forks moving in a non-permissive direction. Replicating chromosomes start segregating into the two halves of the cell early during replication and final segregation occurs after completion of replication. However, the replication and segregation of chromosomes is far from being a simple phenomenon, as replisomes need to overcome several obstacles encountered while replicating the entire genome. These obstacles include, but are not limited to, fork breakage or stalling due to tightly bound proteins, DNA-protein crosslinks and replication-transcription conflicts.

Almost 50 years of intensive research have established profound roles of *E. coli* recombination proteins, required for the repair of DNA double-strand breaks (DSBs), in overcoming these obstacles to allow successful chromosome replication and to preserve genomic integrity (Skalka 1974; Capaldo and Barbour1975a,b; Kuzminov 1995; Kuzminov 1999; Michel *et al*. 2018). Elegant genetic and biochemical experiments, carried out during this period, have revealed intricate details of their enzymatic functions, requirements in the cell, and mutant phenotypes. However, several interesting new observations, pertaining to the impact of DSB repair mutants on genome integrity and propagation, have been reported in the last decade and, accordingly, novel functions have been assigned to the proteins encoded (Rudolph *et al*. 2013; Wendel, Courcelle and Courcelle 2014; Azeroglu *et al*. 2016; Dimude *et al*. 2016; Sinha *et al*. 2017; Dimude, Midgley-Smith and Rudolph 2018; Midgley-Smith, Dimude and Rudolph 2018; Sinha *et al*. 2018; Wendel *et al*. 2018; Midgley-Smith *et al*. 2019). Since these recent discoveries have sometimes led to apparently contradictory interpretations, it is necessary to discuss the experiments and conclusions together, while trying to understand the phenomena. It is also important to discuss these recent observations in the context of the wealth of information generated about these proteins in previous research and what is known about how recombination underpins DNA replication in other bacteria, bacteriophages and archaea. Here, we shall first recall the important functions of these proteins, their mutant phenotypes, and then discuss whether these known functions are able to explain the recent observations.

## RECOMBINATIONAL REPAIR PROTEINS RecBCD, RecA, RecFOR, RuvABC AND THEIR CORRESPONDING MUTANT PHENOTYPES

**Figure 1.**
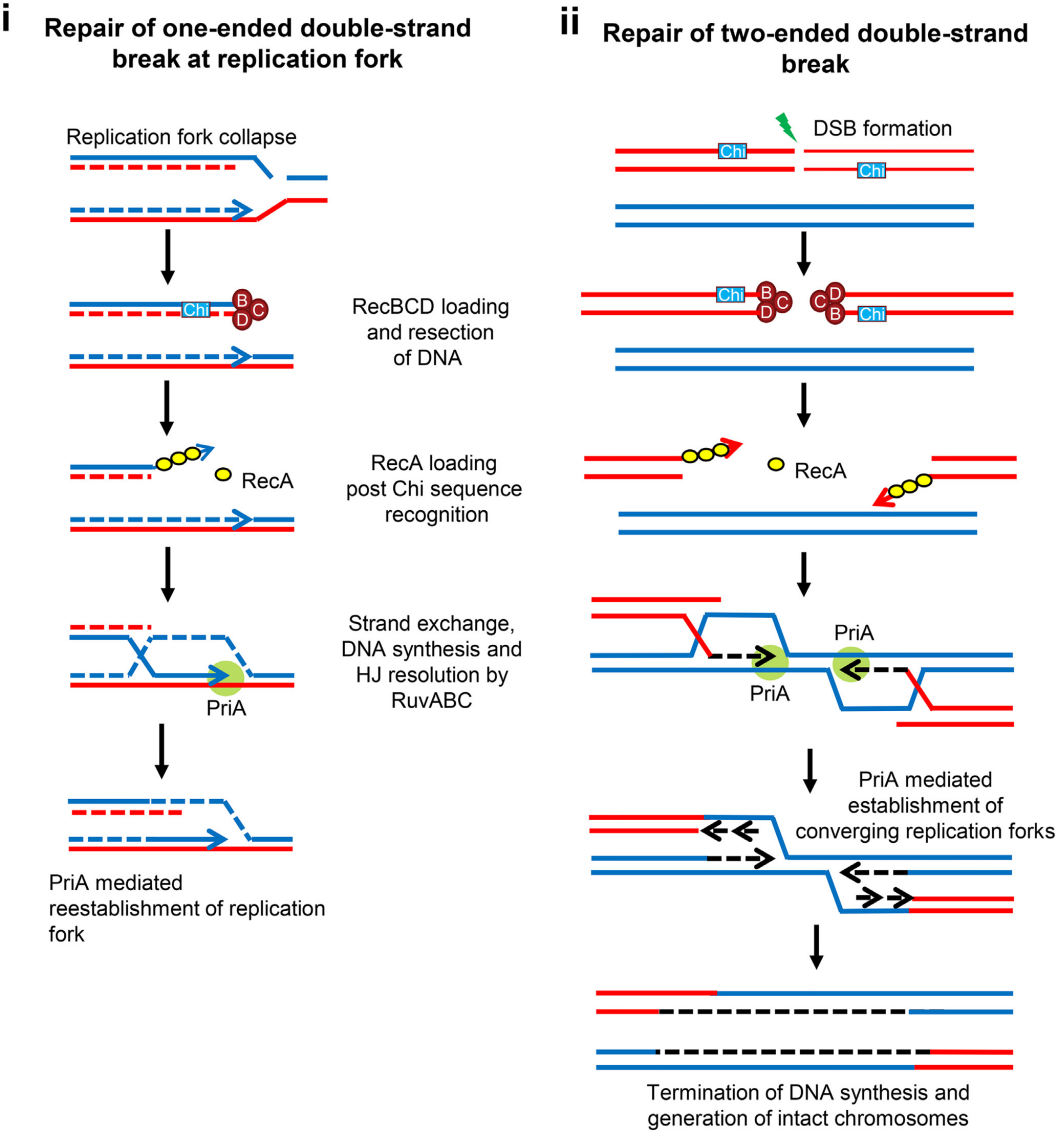
RecBCD-dependent homologous recombinational repair of one-ended and two-ended DSBs. **(i)**, A one-ended DSB is generated when a replication fork encounters a nick in a template strand, leading to replication fork collapse (a broken replication fork). **(ii)**, Direct exposure to ionizing radiation generates a two-ended DSB. Both types of DSBs are unwound and cleaved by RecBCD using its helicase and nuclease activities. After recognizing the regulatory sequence Chi (5’-GCTGGTGG-3’), the properties of RecBCD enzyme change and a 3′-overhang terminated at a Chi sequence is generated. RecBCD then facilitates RecA loading onto this 3′-ssDNA overhang. The RecA-bound ssDNA searches for a homologous duplex DNA sequence, strand-exchange takes place to form a D-loop, followed by Holliday junction branch-migration and resolution by RuvABC proteins. PriA binds to the 3′ paired end(s), facilitates primosome assembly, and allows replication restart. Importantly, recombinational repair of a one-ended DSB leads to re-establishment of a replication fork, whereas two intact chromosomes are the products of two-ended DSB repair. Template strands and nascent strands are shown here as intact and dotted lines respectively. The leading-strand is denoted as a line with an arrowhead to mark the 3′-end.

The RecBCD pathway is essential for homologous recombination mediated DSB repair in *E. coli*. Biochemically, the RecBCD protein complex is an exonuclease (ExoV) that has both 5´-3´ (in RecD) and 3´-5´ (in RecB) helicase activities (Dillingham and Kowalczykowski 2008). RecBCD can only bind to blunt or nearly blunt DNA double-strand ends (DSEs) and, owing to its helicase and exonuclease activities, it unwinds and cleaves DNA simultaneously. Encountering the regulatory ‘Crossover hotspot instigator’ (Chi) sequence (5´GCTGGTGG 3´), RecBCD changes its property from nuclease to recombinase by generating a 3´ overhang onto which it loads RecA (Anderson and Kowalczykowski 1997; Dillingham and Kowalczykowski 2008; Handa *et al*. 2012; Taylor *et al*. 2014). RecA carries out homology searching in an intact homologous template (e.g. the sister chromatid), a Holliday junction is then formed, and this is resolved by RuvABC to form intact recombinants (Fig. 1) (Reviewed in (Kuzminov 1999; Dillingham and Kowalczykowski 2008; Michel and Leach 2012; Smith 2012)). Inactivation of either RecB or RecC abolishes the enzyme's activity, whereas RecD inactivation primarily affects the nuclease activity of the enzyme complex, present in RecB. Accordingly, *recB* and *recC* single mutants are recombination deficient whereas *recD* mutants are recombination proficient. Furthermore, *recB, recC* or *ruvAB* mutants are equally sensitive to exposure to DNA damaging agents such as of UV, ionizing radiation (such as X-rays, gamma rays etc.) or mitomycin C, while a *recD* mutant shows resistance similar to wild-type cells (Lloyd *et al*. 1984; McGlynn and Lloyd 2000; Dillingham and Kowalczykowski 2008). A *recA* mutant is extremely recombination deficient and sensitive to these DNA damaging agents owing to its role in both the RecBCD pathway and the alternative RecFOR pathway (Kuzminov 2011) (discussed below) (Table 1).

**Table1.** Viabilities and recombination frequencies associated with recombination deficient mutants of E. coli

Genotype	Viability (%)	Recombination frequency^a^
Wild type	100	1
Δ*recBCD/*Δ*recB/*Δ*recC*	∼30	0.004
Δ*recA*	∼50	0.00006
Δ *recBC recA*	∼18	0.00006
Δ*recD*	100	1
Δ*recA recD*	∼50	0.00004
Δ *sbcB sbcD*	∼96	
Δ *recBC sbcB sbcC*	∼ 45	0.13
Δ *recBC sbcB sbcC recA*	∼12	
Δ *recBC sbcB sbcC recF*	∼13	0.002
Δ *sbcB sbcD recA*	∼19	
Δ *recF*	∼71	0.5
Δ *recA recF*	∼46	
Δ *recBCD recF*	∼30	
Δ *recG*	∼90	0.3

a Value denotes conjugational recombination frequency.

(Clark and Margulies 1965, Horii and Clark 1973; Lovett, Luisi-DeLuca and Kolodner 1988; Lloyd 1991; Miranda and Kuzminov 2003; Zahradka *et al*. 2006; Zahradka *et al*. 2009).

### Viability

To discover the requirement of RecBCD in *E. coli* cells, grown under standard laboratory conditions (in the absence of external DNA damaging agents), an elegant experiment was performed to check colony forming abilities of equal numbers of cells (counted under the microscope) from wild type and mutants (Miranda and Kuzminov 2003). Interestingly, only ∼30% of Δ*recBCD*, ∼50% of Δ*recA*, and ∼20% of Δ*recBCD* Δ*recA* cells could form colonies on plates, suggesting RecA-dependent and RecA-independent roles of the RecBCD complex (Table 1). Further experiments from this study showed that the RecA-independent requirement for RecBCD is dependent on its exonuclease activity. Similar losses of viability for these mutants were also reported in an earlier study (Capaldo, Ramsey and Barbour 1974). These observations led to the proposal that a sub-population of growing *E. coli* cells experience the formation of replication dependent DSBs that can be reset by RecBCD-RecA mediated recombination or, in the absence of RecA, the broken chromosomal ends can be degraded by RecBCD exonuclease activity to generate intact chromosomes. Indeed, this proposal also explains earlier reports showing that Δ*recA* cells exhibit an ‘asynchrony phenotype’ containing one or three chromosome forming units instead of the normal two or four, due to selective degradation of individual chromosomes (Skarstad and Boye 1993). Interestingly, it was also discovered that it is partially replicated chromosomes and not fully replicated chromosomes that are prone to RecBCD mediated degradation suggesting replication dependent DSB formation (Skarstad and Boye 1993). Additionally, 5–10% of exponentially growing *recA* mutant cells were found to be anucleate due to ‘reckless’ DNA degradation and that degradation of the entire genome occurs, which is suppressed by inactivation of *recBCD* (Capaldo and Barbour1975a,b; Zahradka *et al*. 2009).

### Fork Reversal

RecA independent RecBCD essentiality was also observed in various replication mutant conditions such as *rep*, *priA* or mutants affected for different subunits of the holoenzyme DNA polymerase III (HE Pol III). In these mutant conditions, processing of arrested replication forks generates DNA double-strand ends (DSEs) via replication fork reversal (RFR). RecBCD is essential to repair these reversed forks, either by RecA mediated recombination or RecA-independent degradation processes (recently reviewed in (Michel *et al*. 2018)). RFR also results from replication forks encountering DNA lesions (Fujiwara and Tatsumi 1976; Higgins, Kato and Strauss 1976; McGlynn and Lloyd 2000; Khan and Kuzminov 2012) and has been observed in bacteria growing under stress conditions (Schapiro, Libby and Fang 2003; Sinha *et al*. 2013).

### RecBCD suppressors

Inactivation of the 3´-5´ exonuclease ExoI (encoded by the *xonA* gene, also known as *sbcB* for suppressor of *recBC*) was shown to suppress the recombination deficiency and DNA damage sensitivity of *recBC* mutant cells (Kushner *et al*. 1971; Kushner, Nagaishi and Clark 1972; Templin, Kushner and Clark 1972). Subsequently, it was discovered that these suppressor cells had another mutation in the *sbcC* gene, so in fact the genotype of these cells was *recBC sbcB sbcC* (lacking the activities of RecBCD, ExoI and SbcCD) (Lloyd and Buckman 1985). The SbcCD complex can cleave hairpin DNA molecules *in vitro* and *in vivo* (Leach, Okely and Pinder 1997; Connelly, Kirkham and Leach 1998; Eykelenboom *et al*. 2008). Recombination in these suppressor cells is dependent on another recombination pathway known as the RecF pathway that requires the products of the *recA, recFOR, recQ, recJ*, and *ruvABC* genes. Remarkably, conjugation and transduction efficiency mediated by the RecF pathway in *recBC sbcB sbcC* cells is as efficient as that of wild-type cells (Horii and Clark 1973; Lloyd, Picksley and Prescott 1983; Lovett and Clark 1983; Lloyd and Buckman 1985). It was proposed that elimination of the 3´-5´ exonuclease activity of exonuclease I (by the *sbcB* mutation) in a *recBC* strain allows the survival of a 3′ ssDNA tail that can be used by RecFOR proteins to load RecA and initiate recombination (Kushner *et al*. 1971; Zahradka *et al*. 2006a). The *sbcC* mutation is understood to further enhance the survival of this 3′ ssDNA tail by inactivating the exo-endonuclease activity of SbcCD, which is able to remove single-strand DNA overhangs by cleaving within the adjacent dsDNA (Connelly, de Leau and Leach 1999).

Interestingly, although inactivation of *sbcB* and *sbcC* almost completely suppresses the recombination deficiency of *recBCD* mutants, these suppressors can only partially rescue the viability of these cells. *recBC sbcB sbcC* cells show improved viability up to ∼40%–45%, compared to *recBCD* cells, which are only ∼30% viable. Nevertheless, the viability of *recBC sbcB sbcC* cells is essentially dependent on RecFOR-RecA mediated recombinational repair, as only ∼10% cells of either *recBC sbcB sbcC recA* or *recBC sbcB sbcC recF* can make colonies and these strains are very sick (Table 1; (Zahradka *et al*. 2006a; Zahradka *et al*. 2009)). In agreement with protection of the 3′ ssDNA tail required for RecFOR mediated recombination and viability, the presence of another allele (*sbcB15*) significantly improves viability of *recBC sbcB sbcC* cells. *recBC sbcB15 sbcC* cells are ∼75% viable. It has been proposed that the SbcB15 protein binds to the 3′ ssDNA tail and protects it from other nucleases (Zahradka *et al*. 2006a; Thoms *et al*. 2008).

**Figure 2.**
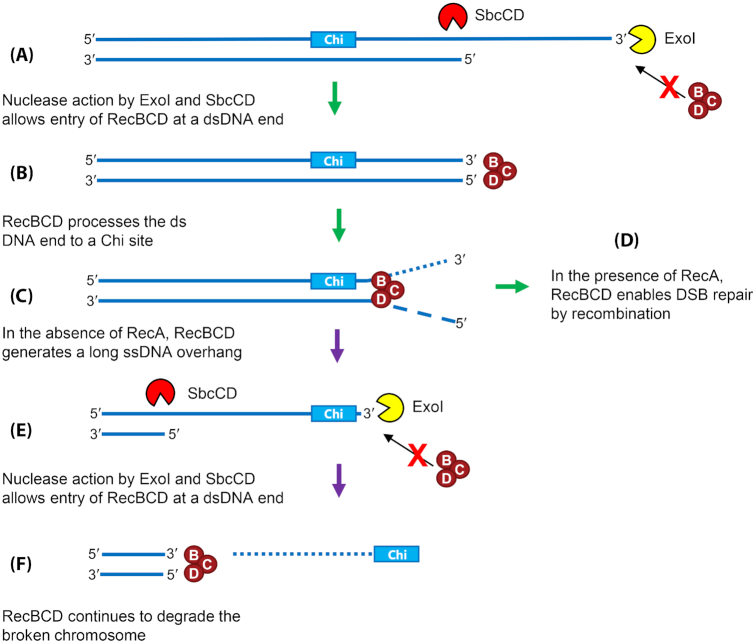
Action of ExoI and SbcCD nucleases facilitates RecBCD-mediated DSB repair by recombination in *recA*^+^ cells and facilitates DNA degradation by RecBCD in *recA* mutant cells. RecBCD enzyme can only bind and initiate unwinding and degradation of DNA that contains a blunt or nearly blunt DSE. Exonuclease I (the product of the *sbcB* gene, also known as *xonA*) and SbcCD both contribute to the generation of such ends that can bind RecBCD. This enables productive DSB repair by recombination, in the presence of RecA (from **A** to **D**) or degradation of a broken chromosome, in the absence of RecA (from **C** to **F**).

The *sbcB sbcD* mutant combination adversely affects the recombination efficiency of the RecBCD complex (in RecA^+^ cells) suggesting that eliminating ExoI and SbcCD activities makes RecBCD activity inefficient (Thoms and Wackernagel 1998; Seigneur, Ehrlich and Michel 1999). Accordingly, the combination of *sbcB* and *sbcD* mutations (in RecBCD^+^ cells) also inhibits the formation of anucleate cells and ‘reckless’ DNA degradation in a *recA* culture, similarly to the effect of a *recB* or *recC* mutation (Zahradka *et al*. 2009). Conversely to the activation of the RecF pathway described above, it was proposed that the action of SbcB and SbcCD proteins, to remove ssDNA overhangs at DSEs to make them blunt, allows efficient loading of RecBCD onto DNA and activation of the RecBCD pathway (Fig. 2A–D). This nucleolytic action of SbcB and SbcCD also becomes important for degradation of the entire chromosome or of a broken chromosomal arm in *recA* mutant cells. We suggest that frequent encounter of Chi sites on the genome of an *sbcB sbcCD* mutant will inactivate RecBCD and leave a long tail of post-Chi 3´-ended ssDNA that needs to be degraded by 3´-5´ exonucleases to allow reloading of RecBCD (Fig. 2c-f). Therefore, in a *sbcB sbcCD* strain, RecBCD cannot rescue cells by degrading a large part of a broken chromosome and it has to rely on RecA-loading for the recombination pathway, which is probably the reason for *sbcB sbcCD recA* (RecBC^+^) strain being only ∼20% viable (similarly to the *recBCD recA* strain) (Table 1; (Thoms and Wackernagel 1998; Thoms *et al*. 2008, Zahradka *et al*. 2009). This inactivation of RecBCD in an *sbcB sbcCD* mutant is different from the inactivation of RecBCD by Chi in the presence of ExoI and SbcCD, which is dependent on RecA and is likely to be caused by titration of RecBCD molecules engaged in Chi-dependent DSB repair events (Kuzminov, Schabtach and Stahl 1994; Kuzminov and Stahl 1997; Thoms and Wackernagel 1998).

### Plasmid maintenance

The requirements for recombination proteins in plasmid multimerization, HMW (High-molecular-weight) DNA formation, and their impact on plasmid stability have been studied extensively. For θ–type plasmids, RecA was shown to be essential for HMW DNA formation suggesting that the HMW structures are formed by intermolecular homologous recombination, whereas for rolling-circle replicating (rcr) plasmids RecA facilitates HMW formation by protecting them from RecBCD mediated degradation (Cohen and Clark 1986; Silberstein and Cohen 1987; Niki *et al*. 1990; Dabert, Ehrlich and Gruss 1992; Dabert, Ehrlich and Gruss 1992; Kuzminov, Schabtach and Stahl 1994). Formation of HMW DNA by plasmid multimerization is responsible for the instability of plasmids due to its impact on copy number, resulting in improper segregation (Biek and Cohen 1986; Niki *et al*. 1988; Niki *et al*. 1990; Wendel, Courcelle and Courcelle 2014; Hamilton *et al*. 2019).

HMW DNA of mini-F and pBR322 plasmids is accumulated in *recD* and in *recBC sbcB* mutants. Both of these strains lack exonuclease V activity but are recombination proficient, by RecBC in the former and by RecFOR in the latter strain (Cohen and Clark 1986; Niki *et al*. 1990). It was proposed that when a θ replicating plasmid is converted to rolling-circle replication either by endonuclease activity or by a fork encountering a nick, the newly generated tail could be degraded by RecBCD to facilititate formation of a monomer. However, in recombination proficient, exonuclease V deficient *recD* and *recBC sbcB* mutants, the tail can recombine with an intact circular molecule to make a HMW multimer lacking a DSE (Nussbaum and Cohen 1988). Such molecules have been described as ‘pince-nez chromosomes’ (Khan *et al*. 2016) and detected as exonuclease-resistant HMW DNA in a *recBC sbcB sbcC* mutant that is recombination proficient (*recA*^+^*recF*^+^) (Kusano, Nakayama and Nakayama 1989). An important point is that HMW DNA formation in wild type cells is dependent on the presence or absence of properly oriented Chi sites that will enhance intermolecular recombination by RecBCD and RecA proteins (Dabert, Ehrlich and Gruss 1992; Kuzminov, Schabtach and Stahl 1994; Smith 2012).

Plasmid multimerization and instability in a *recBC* mutant have recently been re-examined and an attempt has been made to extrapolate from the behavior of plasmids to that of the chromosome (Wendel, Courcelle and Courcelle 2014; Hamilton *et al*. 2019). However, fork breakage, which triggers sigma replication of chromosomes in a *recBC* mutant, occurs in 18% of cells on a 4.6 Mb chromosome (discussed later in this review). This corresponds to one break for every 25.5 Mb of DNA replicated. Clearly, such events occurring on a 5 kb plasmid present at 50–80 copies per cell would not be frequent enough to cause the observed growth impairment of a *recBC* mutant following transformation with a lambda-origin plasmid (Hamilton *et al*. 2019). Furthermore, the impact of nucleases on a 5 kb plasmid and on the chromosome are expected to be very different. A nuclease such as RecBCD, which acts at 1 kb per second, would totally degrade a 5 kb plasmid in 5 seconds, preventing any possibility of DSB repair, whereas in the chromosome it would on average take less than 5 seconds to reach a Chi site and initiate recombination, having degraded less than 0.1% of the genome.

## DNA DOUBLE-STRAND BREAK REPAIR IN GRAM-POSITIVE BACTERIA

DSB repair processes in Gram-positive bacteria exhibit both similarities and differences to the processes in *E. coli*, described above. Nevertheless, the chronology of events following DSB generation is broadly similar (i.e. recognition of the broken ends and processing to generate a 3′ ssDNA tail, loading of RecA onto this ssDNA, followed by strand exchange, branch migration and resolution of Holliday junctions, and replication restart). However, despite having similar recombination machineries, there are structural and functional differences in the individual proteins encoded by *E. coli* and the Gram-positive bacterium *Bacillus subtilis*.

A well characterized feature of DSB repair in *B. subtilis* is the formation of a ‘repair center’ (RC), which is marked by early association, and stabilization of DNA ends by RecN, a member of the SMC (structural maintenance of chromosomes) family of proteins (Ayora *et al*. 2011). This RC could be similar to the RC of eukaryotic systems, which facilitates repair of multiple DSBs at a single location, and is characterized by the presence of MRE11-Rad50 (also a member of the SMC family of proteins) (Lisby, Mortensen and Rothstein 2003). The end-processing reaction is carried out by the helicase-nuclease complex AddAB (a functional analogue of the RecBCD complex) to generate a 3′-ssDNA overhang for RecA loading. The AddA subunit is a distant homologue of RecB, containing an N-terminal helicase domain and a C-terminal nuclease domain, whereas the AddB subunit has a second nuclease domain, at its C-terminus, not present in RecBCD. Similarly to the *E. coli* RecBCD complex, AddAB is regulated by a Chi site (5′ AGCGG 3′). However, in contrast to the RecBCD complex, there is no evidence of RecA loading by AddAB. Instead, RecOR (part of the RecFOR complex) has been shown to load RecA (Dillingham and Kowalczykowski 2008; Lenhart *et al*. 2012). RecA then carries out strand invasion and Holliday junctions are formed. Branch migration is mediated by RuvAB and Holliday junctions are resolved by the RecU endonuclease to reestablish replication forks.

Similarly to *E. coli*, another repair pathway includes the RecFOR proteins, RecQ helicase, and RecJ exonuclease. In this pathway, the 5′ end at a DSB can be degraded by RecJ, in concert with a RecQ helicase, and is followed by binding of SsbA protein to ssDNA. The RecFOR complex promotes the disassembly of SsbA and the loading of RecA. In contrast to *E. coli*, the ExoI protein (SbcB) is absent in *B. subtilis*, while the RecFOR pathway is constitutively active. This implies that a multistep repair process, including multiple redundant protein functions, can promote DSB repair in *B. subtilis* (Ayora *et al*. 2011; Lenhart *et al*. 2012).

Inactivation of AddB and RecJ, in the *addAB recJ* double mutant, shows a synergistic loss of viability and increased sensitivity towards DNA damaging agents, similar to that of a *recA* single mutant (Sanchez *et al*. 2006). Fluorescently tagged RecQ and RecJ were observed to colocalize with the replisome in normal growing cells, in the absence of any exogenous DNA damage, suggesting that their active roles may be in replication fork maintenance (Lecointe *et al*. 2007; Costes *et al*. 2010). Interestingly, the DNA damage response also determines the coupling between sporulation and DNA replication, sporulation being inhibited in a *recA*-dependent manner following DNA damage (Ireton and Grossman 1992). Additionally, RecA, RecO, AddAB, and RecU were shown to be required for replication restart, leading to efficient survival, following replication-transcription conflicts in *B. subtilis* (Million-Weaver, Samadpour and Merrikh 2015).

The extremely radiation resistant Gram-positive bacterium *Deinococcus radiodurans* is a curious case as it lacks any functional homolog of AddAB (or RecBCD). However, it does contain homologs of the RecF pathway proteins including RecFOR, RecJ, RecQ and RecA. Interestingly, RecJ is an essential protein for cell viability in this organism (Bentchikou *et al*. 2010). *Deinococcus radiodurans* can maintain 100% cell viability at a 10–20 fold higher radiation dose than is required to kill *E. coli* (Daly *et al*. 2004). This amazing survival potential is due to rapid reconstruction of an intact functional genome from shattered genomic fragments by an extended synthesis-dependent strand annealing (ESDSA) reaction and homologous recombination. ESDSA requires chromosomal fragments with overlapping sequences that are then used both as primers and as template for complementary strand synthesis (Nassif *et al*. 1994; Zahradka *et al*. 2006b) and is facilitated by the existence of multiple copies of the genome in a single cell. As synthesis of an intact genome is dependent on RecFOR, RecA, and RecJ proteins, *recFOR* or *recA* mutants are extremely radiation sensitive (Zahradka *et al*. 2006b; Bentchikou *et al*. 2010).

## STABLE DNA REPLICATION (SDR)

The term Stable DNA Replication (SDR) was introduced to describe chromosome replication that is *oriC* and DnaA independent (Kogoma and Lark 1970; Kogoma 1978; Kogoma 1986; Kogoma 1997). *E. coli* mutants were isolated that can constitutively express SDR activity and this was termed ‘constitutive SDR (cSDR)’ (Kogoma 1978; Kogoma and von Meyenburg 1983). These cSDR mutants carried mutations in the *rnhA* gene, which encodes RNaseHI, an RNase that removes the RNA strand from RNA-DNA hybrids (R-loops). SDR can also be induced by repair of DSBs introduced either by UV treatment, thymine starvation, or by nalidixic acid treatment (Kogoma and Lark 1975; Kogoma, Torrey and Connaughton 1979; Magee and Kogoma 1990). This type of SDR was called ‘induced SDR (iSDR)’ and involves RecA-mediated recombination-dependent replication (Kogoma and Lark 1975; Kogoma 1986; Magee and Kogoma 1990; Asai and Kogoma1994a). iSDR was measured as replication efficiency post DNA damage in the presence of rifampin and chloramphenicol to block *oriC* initiation (Magee *et al*. 1992). Both iSDR and cSDR require functional RecA protein (Lark and Lark 1979; Kogoma *et al*. 1985; Magee and Kogoma 1990).

### cSDR

*rnhA* inactivation supports growth of *E. coli* cells deleted for *oriC* or inactivation of DnaA protein in minimal medium but not in rich medium (Kogoma and von Meyenburg 1983; Kogoma 1997). Contrary to iSDR, cSDR is sensitive to rifampin, which inhibits transcription. Thus, it was established that cSDR originates from R-loops, where the pairing of ssRNA within a dsDNA molecule is recognized and processed by PriA-mediated replication restart. Unlike iSDR, cSDR does not involve DSBs or SOS induction, thus it is not error prone. Using marker frequency analysis, five sites were shown to have higher copy number in *rnhA* mutant cells. They were named *oriK* sites. Interestingly, two of them were found to be near *terC* (de Massy, Fayet and Kogoma 1984).

### iSDR

RecBCD- and RecA-mediated recombination is necessary for iSDR, indicating that D-loop mediated replication occurs, which has the potential to replicate a large part of the chromosome (Magee and Kogoma 1990; Magee *et al*. 1992; Asai *et al*. 1993; Asai and Kogoma1974a,b,c; Asai, Bates and Kogoma 1994; Asai, Imai and Kogoma 1994). Surprisingly, marker frequency analysis using Southern hybridization revealed two specific regions with an increase in copy number during iSDR. One was in the *oriC* region (termed *oriM1*) and the other was in the *terC* region (termed *oriM2*) (Magee *et al*. 1992; Asai, Imai and Kogoma 1994). In these studies, iSDR was induced by either thymine starvation, UV irradiation or nalidixic acid treatment. Since these treatments are expected to generate DSBs across the genome (though later studies suggested thymine starvation causes breaks near *oriC* (Kuong and Kuzminov^2012^; Khan and Kuzminov^2019^)), D-loops are expected to be formed at distributed locations and this does not simply explain why Magee *et al*. 1992 observed a copy number peak in the *terC* region. Importantly, the high copy number at *terC* was observed upon treatment with nalidixic acid regardless of the presence or absence of *oriC*, suggesting that it has nothing to do with forks originated at *oriC*.

It might be possible that D-loops and iSDR were generated across the genome but the high copy number was visible in the *terC* region due to blockage of forks at *terC*. Alternatively, the generation of DSBs across the genome and their repair might somehow induce increased copy number at *oriM1* and *oriM2*. However, an attempt to map specific loci that can exhibit *oriM1* and *oriM2* activity in a heterologous system was not successful, suggesting that these increases in copy number are the direct outcome of random chromosome DSB generation and repair by RecBCD-RecA mediated homologous recombination and not due to any sequence-specific origin induction. This was supported by the observation that a *recD* mutation (which induces hyper-recombination) enhances iSDR after thymine starvation and nalidixic acid treatment (Magee & Kogoma 1990; Magee *et al*. 1992; Asai *et al*. 1993). Recently, over-replication at a site of DSB repair has been observed in a *recD* mutant and it has been proposed that the absence of RecD interferes with the coordination of DNA ends during DSB repair, leading to the establishment of divergent DNA replication forks (White *et al*. 2018).

The absence of iSDR in a *recBC* mutant was reversed by inactivation of *sbcA* (which restores homologous recombination), further confirming that iSDR may be simply D-loop mediated replication and the outcome of a functional homologous recombination repair system. iSDR initiation was further enhanced by mutations in genes encoding the Holliday junction resolvase RuvC and the branch migration proteins RuvA or RecG, suggesting that unresolved Holliday junctions might be permissive for iSDR (Asai *et al*. 1993). In conclusion, the reason for the higher copy number specific to the *terC* region in response to the generation and repair of DSBs in iSDR remains unknown (but see section 6 for more details of terminus DNA amplification).

### RecG

Interestingly, continuous origin-independent DNA synthesis and induction of the SOS response was observed in exponentially growing *recG* (and also in *ruvA* and *ruvC*) mutants (Asai and Kogoma1994b). However, experiments were not carried out to discover whether there is any specific origin induction in these cells. RecG is a DNA helicase that has branch migration activity and *recG* mutants are sensitive to DNA damaging agents (Storm *et al*. 1971; Lloyd and Buckman 1991; Kalman, Murphy and Cashel 1992; Lloyd and Sharples 1993). The *recG* mutation is also synthetically lethal with *rnhA* inactivation suggesting that RecG may play a role in R-loop removal (Hong, Cadwell and Kogoma 1995). Thus, continuous DNA synthesis observed in a *recG* mutant could either stem from persistent R-loops or from D-loops accumulated because of the absence of DNA branch migration activity, or both. Accordingly, a *recG* mutant exhibited SDR at a similar level to that of an *rnhA* mutant but only half of it was sensitive to rifampin. R-loop mediated SDR will be sensitive to rifampin, suggesting that at least the other half was dependent on D-loops. Indeed, half of the SDR was sensitive to *recB* mutation, which would block D-loop formation (Hong, Cadwell and Kogoma 1995). Contrary to the *rnhA* mutant, *recG* inactivation could not rescue cells devoid of *oriC* or DnaA, and since the *recG* mutant exhibits SDR at a similar level to that of the *rnhA* mutant (Hong, Cadwell and Kogoma 1995), it was postulated that the mode of SDR (origin usage and direction of replication) in a *recG* mutant may not be suitable to replicate the entire chromosome (Kogoma 1997). We shall discuss this further in section 6 of this review and see how this proposal proved to be correct, as two further mutations are required to make this possible.

### PriA

**Figure 3.**
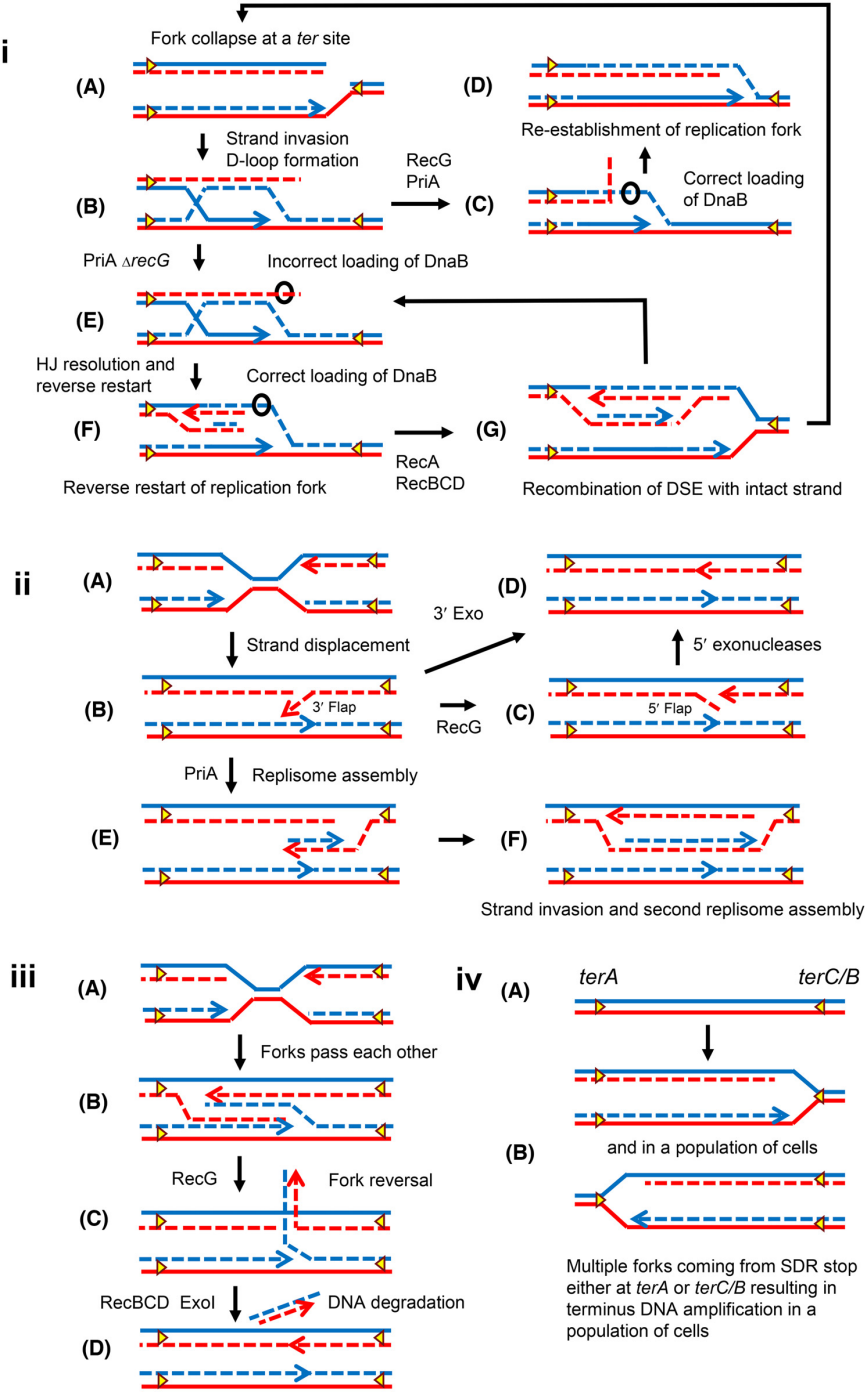
Four models to explain terminus DNA amplification in a *recG* mutant. (i) **Reverse-restart model:** This model proposes concerted action of RecG and PriA in the re-establishment of a correctly oriented replication fork. According to this model, during broken replication fork repair and reassembly, the presence of RecG allows correct PriA-mediated loading of DnaB helicase on the lagging-strand template, potentiating correct replication restart (from **a** to **d**). In the absence of RecG, PriA is no longer correctly directed by RecG and can mistakenly load DnaB on the nascent lagging-strand (**e**). This initiates reverse-restart, via the assembly of a replication fork proceeding in the opposite direction. Meanwhile, PriA can still load DnaB in the correct location setting up a correctly oriented replication fork; (the absence of RecG does not preclude normal DnaB loading, it simply permits incorrect loading) (**f**). The end of the ds-DNA flap that has been generated can invade and recombine with the intact chromosome (RecA-RecBCD mediated recombination). Thus, divergent forks are established causing DNA amplification (**g**). If, during recombination, PriA again loads DnaB incorrectly a new flap can be generated adding to the amplification (from **g** to **e**). If new breaks are generated at one or other of the Tus/*ter* sites, the cycle can be re-initiated adding to amplification (from **g** to **a**). (ii) **3′-flap model:** This model proposes that merging of replication forks in the terminus generates a 3′-flap (from **a** to **b**). RecG binds to this 3′-flap and converts it into 5′-flap to be degraded by 5′ exonucleases to complete termination (from **c** to **d**). In the absence of RecG, the 3′-flap will be used by PriA for primosome assembly and restart of the replication fork (**e**). The end of the ds-DNA flap that has been generated will invade and recombine with the intact chromosome (RecA-RecBCD mediated recombination). Thus, bidirectional forks are established causing DNA amplification (**f**). (iii) **Over-replication model:** This model proposes that merging replication forks can pass each other, leading to amplification of the terminus region (from **a** to **b**). The over replicated region is processed by RecG to make it available for degradation by RecBCD and other exonucleases. In the absence of *recG* or exonucleases, the over replicated region persists. (iv) **Fork-trap model:** This model simply proposes that Tus/*ter* mediated trapping of multiple forks generated by stable DNA replication in a *recG* mutant leads to the observation of terminus DNA amplification (**a-b**). Yellow triangles represent the Tus/*ter* trap; the black circle represents the DnaB helicase. Template strands and nascent strands are shown here as intact and dotted lines respectively. The leading-strand is denoted as a line with an arrowhead representing the 3′-end.

PriA is required for replication restart pathways (Lee and Kornberg 1991; Nurse, Zavitz and Marians 1991; Michel and Sandler 2017; Michel *et al*. 2018). The *priA* null mutant is sick, grows slowly in minimal medium, does not grow in rich medium, and is completely inactive for SDR (Sandler, Samra and Clark 1996; Michel *et al*. 2018). However, PriA helicase activity is not essential for primosome assembly and PriA (K230R) (encoded by the *priA300* allele), which is deficient in ATPase and helicase activity, still supports SDR initiation (Kogoma *et al*. 1996; Dimude *et al*. 2015). Remarkably, inactivation of PriA helicase activity (by the *srgA* or *priA*300 mutations) suppresses the DNA damage sensitivity of *recG* mutants (Al-Deib, Mahdi and Lloyd 1996; Jaktaji and Lloyd 2003). This indicated that PriA and RecG have opposing roles and that the requirement for RecG protein in DSB repair may not be direct. Instead, RecG might prevent undesirable consequences of PriA helicase activity during DSB repair. It has been shown that RecG helps the correct loading of PriA and DnaB for replication restart (Tanaka and Masai 2006) and in the absence of RecG, PriA can load DnaB incorrectly causing replication to restart in the opposite direction (reverse-restart) (Azeroglu *et al*. 2016; Michel *et al*. 2018) (Fig. 3i).

## ORIGIN-INDEPENDENT DNA REPLICATION IN OTHER SYSTEMS

Recombination-dependent DNA replication (RDR) has been observed in several other systems and these can be thought of as analogous to SDR in *E. coli*. Furthermore, RDR can lead to genome replication, even without the need for replication initiation from a fixed origin of replication.

Bacteriophage T4 is the classic model system where RDR was originally proposed and studied (Luder and Mosig 1982; Kreuzer *et al*. 1988). T4 expresses a full set of its own recombination proteins at high level during T4 infections of *E. coli*. In fact, T4 uses D-loop formation as a major mode of DNA replication initiation during late stages of the lytic cycle (Kreuzer and Brister 2010; Liu and Morrical 2010). Initiation of early T4 DNA replication occurs at specific origins (*oriF* and *oriG*) by the formation of R-loops. Since the infecting T4 phage genome is linear and circularly permuted, when this origin-initiated replication reaches the DNA ends, randomly located ssDNA 3′ termini are generated. These 3′ ssDNA ends are then used by the recombinase UvsX (recruited by UvsY to polymerize on gp32-complexed ssDNA) to catalyze strand invasion, either into the other end of the same phage genome, which has the same sequence repeated, or into the middle of another phage DNA (facilitated by the circular permutation of the genome). Strand invasion is followed by D-loop mediated replication initiation and is thus responsible for net genome duplication in the late stages of lytic cycle (Luder and Mosig 1982; Kreuzer *et al*. 1995; Kreuzer and Brister 2010; Liu and Morrical 2010).

Origin independent replication has also been reported in the archaeon *Haloferax volcanii*. This archaeon can survive even after deletion of all four of its replication origins. Interestingly, the deletion of all four origins confers 7.5% faster growth rate than the wild type strain. Nonetheless, the absolute requirement for the recombinase RadA indicates that D-loop/R-loop mediated replication initiation (similar to SDR in *E. coli*) is responsible for chromosomal replication and survival of the origin-deleted *H. volcanii* strain (Ausiannikava and Allers 2017).

## RECENT OBSERVATIONS OF AMPLIFICATION AND DEGRADATION OF DNA IN THE TERMINUS OF *E. coli*MUTANTS

In the last decade or so, a surge of new observations has emerged on the effects of *E. coli* DSB repair mutants on genome integrity and propagation, stimulated in large part by whole genome sequencing methods of marker frequency analysis (MFA). The two most prominent observations are amplification and loss of DNA in the terminus region. A large set of mutants exhibit terminus region amplification, whereas terminus region loss is specific to *recBC* mutants (Dimude *et al*. 2015; Ivanova *et al*. 2015; Dimude, Midgley-Smith and Rudolph 2018; Rudolph, Upton and Lloyd^2009^; Rudolph *et al*.^2009^, ^2013^; Wendel, Courcelle and Courcelle 2014; Courcelle *et al*. 2015; Sinha *et al*. 2017, 2018; Wendel *et al*. 2018; Raghunathan *et al*. 2019. We shall first discuss the evidence and mutant conditions for terminus amplification and then analyze DNA loss separately.

### DNA amplification of the chromosome terminus region

Terminus DNA amplification has been reported in *recG*, *dam*, *exo, rnhA* and *topA* mutants. Given the diversity of these genes, we expect that this will not be an exclusive list of genes implicated in minimizing terminus region amplification. Recently, it has been shown that DSB repair can stimulate terminus region amplification even in a wild type cell (White *et al*. 2020).

#### Amplification in *recG* mutant

When iSDR in the *recG* mutant was reinvestigated using pulsed field gel electrophoresis (PFGE), it was shown to occur all over the genome following UV irradiation (Rudolph, Upton and Lloyd^2009^; Rudolph *et al*.^2009^). This was satisfying as UV was expected to induce distributed DSBs (Khan and Kuzminov 2012), the repair of which could induce randomly distributed D-loop mediated replication. In addition, when the *ori* and *ter* regions were examined by fluorescence microscopy, amplification of these two locations was confirmed upon iSDR, similarly to the previous description of *oriM1* and *oriM2* (Magee *et al*. 1992; Asai, Imai and Kogoma 1994, Rudolph, Upton and Lloyd^2009^; Rudolph *et al*.^2009^). Interestingly, *recG* inactivation delayed the recovery of UV irradiated cells. These cells remained elongated, had much higher numbers of both origin and terminus foci and were affected for chromosome segregation for a long time compared to wild type cells. Nonetheless, this phenotype was also suppressed (or highly reduced) by inactivation of PriA helicase activity (Rudolph, Upton and Lloyd^2009^; Rudolph *et al*.^2009^). Based on these data, a model was proposed to explain persistent high SDR activity in UV irradiated *recG* cells.

According to this model, induced replication forks would meet each other and the replisome of one fork may displace the 3´-end of the oncoming fork generating a 3´ flap. RecG, owing to its preference to unwind a fork substrate with a 5’ end at the fork junction, will bind and convert a 3’ flap to a 5´ flap thereby limiting PriA mediated re-replication in the opposite direction. The 5´ flap will be eliminated by a 5´-3´ ssDNA exonuclease. In the absence of RecG, PriA mediated restart will further generate a DSE substrate for RecBCD-RecA mediated recombination and amplify the region adjacent to the point of fork collision (Rudolph, Upton and Lloyd^2009^) (Fig. 3ii). However, this model assumes PriA mediated restart from a 3´ flap, which has not been documented biochemically. Instead, it has been shown that PriA needs a 3' end paired to the template to initiate replication (Heller and Marians 2005, Bhattacharyya *et al*. 2014, Michel and Sandler 2017). Therefore, PriA mediated restart from a 3´ flap remains hypothetical.

Using whole genome sequencing based MFA analysis, a striking observation was made. An exponentially growing *recG* mutant specifically amplifies the copy number of the terminus region, between *terA* and *terB*, even without any UV treatment (Rudolph *et al*. 2013). This amplification is abolished by inactivation of PriA helicase activity and by inactivation of *recB* suggesting SDR involvement. This SDR dependent amplification in a *recG* mutant allows cells to tolerate and survive the loss of DnaA if two further obstacles are removed: first, in a *tus* mutant outward directed forks can move beyond the terminus region; and second: in a rpoB*35 mutant, which destabilizes transcription complexes, forks can proceed round the chromosome (Rudolph *et al*. 2013). These results established that SDR mediated replication (including its requirement for RecBCD-RecA mediated DSB repair) is efficient in replicating the entire chromosome but is normally limited by replication-transcription collisions, since most of the highly transcribed genes (e.g. rRNA operons) are co-directional with replication forks generated at *oriC*, whereas replication forks generated by SDR have a high chance of moving in the opposite direction (Rudolph *et al*. 2013; Dimude *et al*. 2015). Accordingly, a dnaA46 recG tus rpo* strain (no replication initiation at *oriC*, no RecG, and no blockage by Tus or RNA polymerase of replication forks moving from terminus to origin) grows at the non-permissive temperature (42°C) and its MFA replication profile shows the highest peak at the terminus and the lowest at *oriC*, the opposite to a normal replication profile.

The same model was used to explain this terminus amplification observed in a *recG* mutant as had been proposed to explain high SDR occurring elsewhere on the chromosome upon UV irradiation (Rudolph, Upton and Lloyd^2009^). Notably, it was proposed that, when two forks (normally originated at *oriC*) meet in the terminus region, they generate a 3´ flap and, in the absence of *recG*, this 3´ flap is used by PriA to restart new replication forks moving in opposite directions (Fig. 3ii). Thus, it was suggested that fork collision has the potential to destabilize genomic integrity, while RecG prevents this by converting 3´ flaps into 5´ flaps at fork collision sites. The observation that inactivation of multiple 3´ ssDNA exonucleases (*xseA xonA sbcCD*) also gives a similar MFA replication profile of terminus amplification, despite the presence of RecG was used as further support for the model (Rudolph *et al*. 2013). However, as mentioned above, a 3´ flap is not a known substrate for PriA mediated replication restart. More importantly, the inverted MFA profile (highest at terminus and lowest at origin) cannot simply be explained by forks merging at the terminus, since forks do not originate from *oriC* at the non-permissive temperature in a strain with the *dnaA46* mutation.

Alternatively, a reverse-restart model was proposed to explain *ter* dependent amplification in the *recG* mutant (Azeroglu *et al*. 2016). According to this model, PriA can promote reverse-restart at a Tus/ter barrier in the absence of *recG* (Fig. 3i). This fork moving in the reverse direction will reach the opposite *ter* site and again a new reverse-restart fork will be assembled in the opposite direction. At each step a DSE will be generated that can recombine to generate another fork. This back and forth replication is proposed to amplify the terminus region in *recG* cells. Support for this model was provided by RecA ChIP-seq data showing outward directed RecA binding at *terA* and *terB* sites, demonstrating the presence and location of the predicted DSEs (Azeroglu *et al*. 2016). Furthermore, DNA amplification in a *recG* mutant was observed at the site of DNA double-strand breakage in the *lacZ* locus, distant from the chromosome terminus (Azeroglu *et al*. 2016), raising the possibility that, in the absence of RecG, reverse-restart can potentially occur following attempted recombination at any chromosomal DSE. It was previously shown that forks blocked at *ter* sites are prone to the generation of DSEs (Horiuchi *et al*. 1994).

Two further models have been proposed. In the third model, it was suggested that merging replisomes might cross each other and over-replicate the terminus region (Fig. 3iii) (Wendel, Courcelle and Courcelle 2014). In this model, RecG was hypothesized to reverse one of these forks generating a DSE that was degraded by RecBCD to remove the over-replication. In this way, a *recG* mutant would retain the over-replicated region. Finally, in the fourth model, it was suggested that SDR initiated at random locations resulted in terminus over-representation in MFA simply because of the nature of the Tus/*ter* trap that would not allow replication to proceed across the terminus to a Tus/*ter* block in two directions in a population of cells (Fig. 3iv) (Gowrishankar 2015).

Not all features of these models are mutually exclusive and some data fit some models and not others, while some data seem incompatible with all models as they currently stand. Several key observations need to be accommodated in our understanding: 1. One-ended DSEs accumulate at Tus/*terA* and Tus/*terB* sites in a *recG* mutant and these are the boundaries of the amplified region in a strain with an intact Tus/t*er* system (Azeroglu *et al*. 2016). 2. In a strain harboring two origins (*oriC* and *oriZ*), amplification is only seen at the terminus site although forks meet at two different places on the chromosome (Rudolph *et al*. 2013). 3. Terminus amplification is still visible (although reduced) in *recG* cells harboring a linear chromosome, where forks would never meet (Rudolph *et al*. 2013; Dimude *et al*. 2015). 4. The MFA profile of a dnaA46 recG tus rpo* strain shows a maximum in the terminus and a minimum in the *oriC* region. Observations 1. and 2. suggest that an interaction with the Tus/ter system plays some role in the DNA amplification observed in a *recG* mutant and this correlates with an elevated level of DSEs at these sites. Observation 3. suggests that the meeting of replication forks originating from *oriC* is not required for amplification in a *recG* mutant. Observation 4. suggests that there is something important about the terminus region that determines the initiation of DNA replication in a *recG* mutant even in the absence of a functioning Tus/*ter* system and in the absence of DnaA-dependent initiation of replication at *oriC*.

Further investigation revealed that the amplification observed in a *recG* mutant is not dependent on R-loops, as overexpression of yeast RNaseH1 does not affect the viability of a dnaA46 recG tus rpo* strain (Dimude *et al*. 2015). All of these new and earlier observations clearly suggest that RecG keeps D-loops in check to prevent SDR. Exponentially growing *E. coli* cells are prone to suffer replication-dependent DSBs (Kuzminov 1995, 2001; Miranda and Kuzminov 2003; Michel *et al*. 2018). Repair of these DSBs will initiate D-loop mediated replication, which can be exacerbated in absence of RecG, perhaps by a reverse-restart mechanism and then by replication-transcription collisions; but consequences only become visible in the terminus region due to the presence of *ter* sites, which may contribute themselves to the amplification through stimulating further DNA breaks and driving a precise localization of amplified DNA, while replication-transcription collisions occur all over the chromosome.

#### Amplification in *dam* mutant

Recently the *dam* mutant was also shown to have amplification in the terminus region and to allow deletion of *dnaA* when combined with *tus* and rpo* mutations (Raghunathan *et al*. 2019). As for *recG* mutants, the *priA300* allele abolished this survival advantage suggesting SDR. A crucial role of DinG protein in overcoming replication-transcription collisions was also reported when cells are replicating by SDR in a *dam* mutant. SDR initiated in a *dam* mutant is due to DSBs generated by the mismatch repair pathway, as inactivation of either *mutH* or *mutL* or *mutS* abolishes the growth advantage conferred by the *dam* mutation to *dnaA*-deleted cells. Surprisingly, DSBs generated by exposure of the dnaA tus rpo* strain to sub-lethal doses of DNA damaging agents does not allow them to tolerate loss of *dnaA*. Similarly, SbcCD mediated cleavage of a long palindromic sequence in the *lacZ* gene was unable to support growth of the dnaA tus rpo* strain.

#### Amplification in multiple *exo* mutant

Inactivation of at least two exonucleases (*xonA* and *sbcCD* or *xseA*) causes mild amplification of the terminus area and allows dnaA46 tus rpo* xonA sbcCD and dnaA46 tus rpo* xonA xseA strains to grow at the non-permissive temperature (Rudolph *et al*. 2013, Wendel, Courcelle and Courcelle 2014, Wendel *et al*. 2018, Midgley-Smith *et al*. 2019). According to the 3´ flap model (Fig. 3ii) (Midgley-Smith *et al*. 2019), these exonucleases are required to degrade the 3´ flap generated after fork collision in the terminus, so in their absence the 3´ flap will be used by PriA to restart replication, similarly to what was proposed for the *recG* mutant. Nevertheless, in contrast to the *recG* mutant, the amplification seen in these mutants was much weaker despite the suppression of *dnaA* being efficient (Midgley-Smith *et al*. 2019). This precludes making a strong link between the extent of amplification in the terminus and suppression of the *dnaA* requirement for viability but supports the hypothesis of a general upregulation of SDR activity across the genome, terminus amplification being a secondary effect visible due to the fork-trap.

The other model put forwarded to explain terminus amplification in a multiple exonuclease mutant is the replication fork bypass model. Here, ExoI and SbcCD are proposed to process the intermediate generated by RecG to allow its degradation by RecBCD enzyme complex for completion of replication (Fig. 3iii) (Wendel, Courcelle and Courcelle 2014, Wendel *et al*. 2018). However, this model does not explain the survival of a *dnaA* mutant by inactivation of these exonucleases.

#### Amplification in *rnhA* mutant

As previously noted for *oriKs*, whole genome sequencing based MFA analysis of the *rnhA* mutant revealed multiple peaks, including a specific peak in the terminus region (Maduike *et al*. 2014; Dimude *et al*. 2015). In contrast to the *recG* mutant, this peak was very small. Importantly, the *rnhA dnaA tus* triple mutant showed peaks in the regions flanking *terA-terB* (and not in between) suggesting that the peak observed in the fork trap region is due to the blockage of forks coming from outside of this region rather than initiated there (Maduike *et al*. 2014). This gives an informative perspective that a clear peak observed between *terA* and *terB* does not necessarily imply phenomena initiated there. Instead, amplification can appear there due to forks being captured by the fork trap. Despite robust attempts to map specific locations of *oriKs* these have remained elusive and the question needs further investigation (de Massy, Fayet and Kogoma 1984; Nishitani, Hidaka and Horiuchi 1993; Horiuchi *et al*. 1994; Kodama *et al*. 2002; Maduike *et al*. 2014). Regardless of the positions of *oriKs*, it is clear that they originate from R-loops and they can support growth of cells upon inactivation of DnaA. This tolerance becomes more effective by additional mutations of *tus* and rpo*, indicating that forks move in the opposite direction to normal replication. Therefore, they are susceptible to replication-transcription collisions and blockage at *ter* sites, when attempting to replicate the entire chromosome (Dimude *et al*. 2015; Ivanova *et al*. 2015).

#### Amplification in *topA* mutant

The replication profile of *topA topB* null cells (mutant for Topo I and Topo III topoisomerases) clearly shows strong DNA amplification between *terA* and *terB* in addition to three smaller peaks outside terminus (Brochu *et al*. 2018). These cells are very sick and carry compensatory mutations in *gyrB* (DNA gyrase). The terminus amplification and severe growth defects of *topA topB* null cells were suppressed by RNase HI overproduction suggesting the involvement of R-loop mediated cSDR in this strain (Brochu *et al*. 2018; Drolet and Brochu 2019). Indeed a high amount of R-loops and of cSDR were observed in *topA topB* null cells and the growth phenotype could be suppressed either by RNase HI overproduction, *recA* mutation (RecA is required to repair the resulting double-strand breaks) or *dnaT* mutation (required for replication restart) (Masse and Drolet 1999; Martel *et al*. 2015; Kouzminova, Kadyrov and Kuzminov 2017; Brochu *et al*. 2018; Drolet and Brochu 2019).

### Conclusions from DNA terminus amplification studies

Classical and recent studies point to D-loops and R-loops being implicated in SDR and suggest that SDR is intimately associated with terminus DNA amplification. However, the formation of a D-loop is not sufficient to set up SDR since most D-loops are expected to be resolved to restore normal DNA replication (e.g. one-ended DSB repair of a broken replication fork) or an intact chromosome (e.g. two ended DSB repair at the site of ionizing radiation or similar damage) (Fig. 1). What is needed for SDR initiated at a D-loop is the establishment of a replication fork proceeding around the chromosome in the reverse direction to a normal fork. This implies some sort of abnormal reaction. One such reaction is the over-replication of a section of DNA prior to D-loop formation. This is the case for the 3’ flap model (Rudolph, Upton and Lloyd^2009^; Rudolph *et al*. 2013) and the reverse-restart model (Azeroglu *et al*. 2016). Both of these models propose that the initial over-replication event occurs prior to D-loop formation and that the D-loop creates a new replication bubble with forks that can proceed in both directions round the chromosome. The lack of an abnormal reaction leading to a reverse orientated fork is presumably the reason why DSBs generated by DNA damaging agents do not support the growth of a ΔdnaA tus rpoB* strain (Raghunathan *et al*. 2019). SbcCD dependent cleavage of a long DNA palindrome in the *lacZ* gene was shown to generate a two-ended DSB (Eykelenboom *et al*. 2008) that can be processed in 50% of cases to a broken replication fork (Cockram *et al*. 2015). Since both of these structures are repaired without setting up reversely oriented replication forks, palindrome cleavage is also unable to rescue the absence of DnaA in ΔdnaA tus rpoB* strain (Eykelenboom *et al*. 2008; Raghunathan *et al*. 2019). Presumably D-loops set up in *recG*, *dam* and multiple *exo* mutants have the characteristic of enabling replication forks to be established in the reverse chromosomal direction allowing the strains to tolerate the absence of *dnaA*, provided two major obstacles of replication-transcription collision and Tus/*ter* blockage are removed by rpo* and *tus* mutations respectively.

Random replication fork dependent DSBs (∼18%; reviewed in (Michel *et al*. 2018)) augmented by breaks at Tus/ter blocks could be responsible for SDR initiation in the *recG* mutant, whereas MutHLS mediated DSBs associated with replication forks are required to support SDR in the *dam* mutant (Raghunathan *et al*. 2019). The reverse-restart observed in a *recG* mutant can explain why random fork breakage can lead to SDR. In addition, in a small proportion of cells where a fork stalls at a Tus/ter barrier in the *recG* mutant this can lead to a ping-pong amplification of the terminus region via the same reverse-restart reaction. Cells undergoing this ping-pong reaction may be the small subpopulation (∼2%) of the *recG* mutant that are hyper filamented and contain substantially increased numbers of replisomes, when visualized as YPet-DnaN foci (Midgley-Smith *et al*. 2018). Terminus amplification seen in the *recG* mutant is higher due to this active ping-pong mechanism, whereas it is lower in *rnhA*, *dam* or *exo* mutants, where the terminus trap may simply block all forks originated by SDR, as proposed previously (Gowrishankar 2015). The event that precipitates SDR in the multiple *exo* mutant is unknown but this may be where 3′ flaps play a role. It is important to note that the reverse-restart and 3′ flap models are not mutually exclusive, if relevant to different situations. However, as we have discussed earlier, PriA cannot restart replication from a 3′ flap since it needs 3' end paired to the template to initiate replication. Instead, a 3′ flap (3′ ssDNA) might simply be used for RecA loading and strand invasion and then PriA assembly can restart bidirectional replication.

Moreover, if 3′ flaps are formed under certain mutant conditions, we consider it unlikely that they are generated by the collision of two DNA replication forks, as it has been shown that the replicative helicase DnaB does not unwind a 3′ end when the DNA strand encountered is paired to its template. Instead, DnaB encircles the two strands and moves past the 3′ end while leaving it bound to its template (Kaplan 2000; Kaplan and O'Donnell 2002). This biochemical observation provides a simple mechanism to avoid problems when DNA replication forks meet and it should be noted that any problems, which might be caused by replication fork collisions, are currently hypothetical. In fact, any explanation based on the collision of replication forks cannot satisfactorily explain DNA amplification in a *recG* mutant, as this is still observed in a linear chromosome where replication forks do not meet (Rudolph *et al*. 2013; Dimude *et al*. 2015).

The DNA amplification observed in *rnhA* and in *topA* mutants is due to R-loops. It is still not understood what determines the frequency, spatial distribution and stability of the R-loops in the absence of RNaseHI. Nevertheless remarkably, these R-loops are robust enough to support growth of a DnaA inactivated strain even without *tus* or rpo* alleles.

### DNA degradation of the chromosome terminus region

Whole genome sequencing based MFA analysis has revealed DNA loss in the chromosome terminus area of a *recBC* mutant with a maximum loss in the *dif*/*terC* region (Rudolph *et al*. 2013). The first model proposed to explain the DNA loss in a *recBC* mutant suggested that when replication forks meet, in the terminus region, they tend to by-pass each other and over replicate the DNA (Wendel, Courcelle and Courcelle 2014). The model proposed that RecG facilitates unwinding of this over-replicated region (essentially a fork reversal reaction) and that exonucleases ExoI and SbcCD process this region leading to its degradation by RecBCD (Fig. 3iii) (Wendel, Courcelle and Courcelle 2014; Wendel *et al*. 2018). The degradation of this over-replicated region by the RecBCD complex was proposed to facilitate termination of replication and in the *recBC* mutant erroneous degradation of this over-replicated region by other exonucleases would lead to the observed DNA loss (Wendel, Courcelle and Courcelle 2014; Courcelle *et al*. 2015).

Subsequent analysis and experimental work have refuted this first model. Some of the initial studies, showing a maximum DNA loss in the *dif*/*terC* region of a *recBC* mutant (Wendel, Courcelle and Courcelle 2014), were carried out in a strain (W3110) that carries an inversion between *rrnD* and *rrnE* (flanking the origin of replication) that makes the left replichore ∼200 kb shorter than the right replichore predicting termination at *terA* rather than at *dif/terC*. DNA loss focused at *dif*/*terC* was also seen in a *recBC* mutant derivative of a strain harboring an ectopic *ter* site that generates a new fork trap close to *terA*. In this strain, essentially all forks terminate at this new fork trap thus little or no replication terminates at *dif*/*terC*. However, DNA loss still occurs around *dif*/*terC* (Sinha *et al*. 2017). DNA loss due to replication termination was further weakened by observing DNA loss in a *recBC* mutant with an extra origin of replication, *oriZ*. In *recBCD*^+^ cells with two origins of replication and *tus* inactivation (an *oriC oriZ tus* strain), MFA analysis showed that the replication termination point was ∼600 kb away from *dif*/*terC*, as expected. However, *recBC* inactivation in this strain again showed DNA loss exactly at the same point (*dif*/*terC*), clearly showing that the phenomenon is spatially and mechanistically independent of replication termination (Dimude, Midgley-Smith and Rudolph 2018).

RecA ChIP-seq analysis in the terminus of wild type cells was performed to check if any DSBs could be detected in the chromosome terminus under the growth conditions where terminus DNA loss was observed in the *recBC* mutant. Such RecA binding would be expected according to this first model, as RecBCD action would lead to RecA loading following encounter with an appropriately oriented Chi site. However, no RecA binding could be detected arguing against the first model and, more surprisingly, arguing against any model proposing that the terminus-localized DSBs (or DSEs) are formed in wild type cells. Instead, the observation made it clear that these terminus-localized DNA breaks are only generated in the *recBC* mutants (Sinha *et al*. 2017).

**Figure 4.**
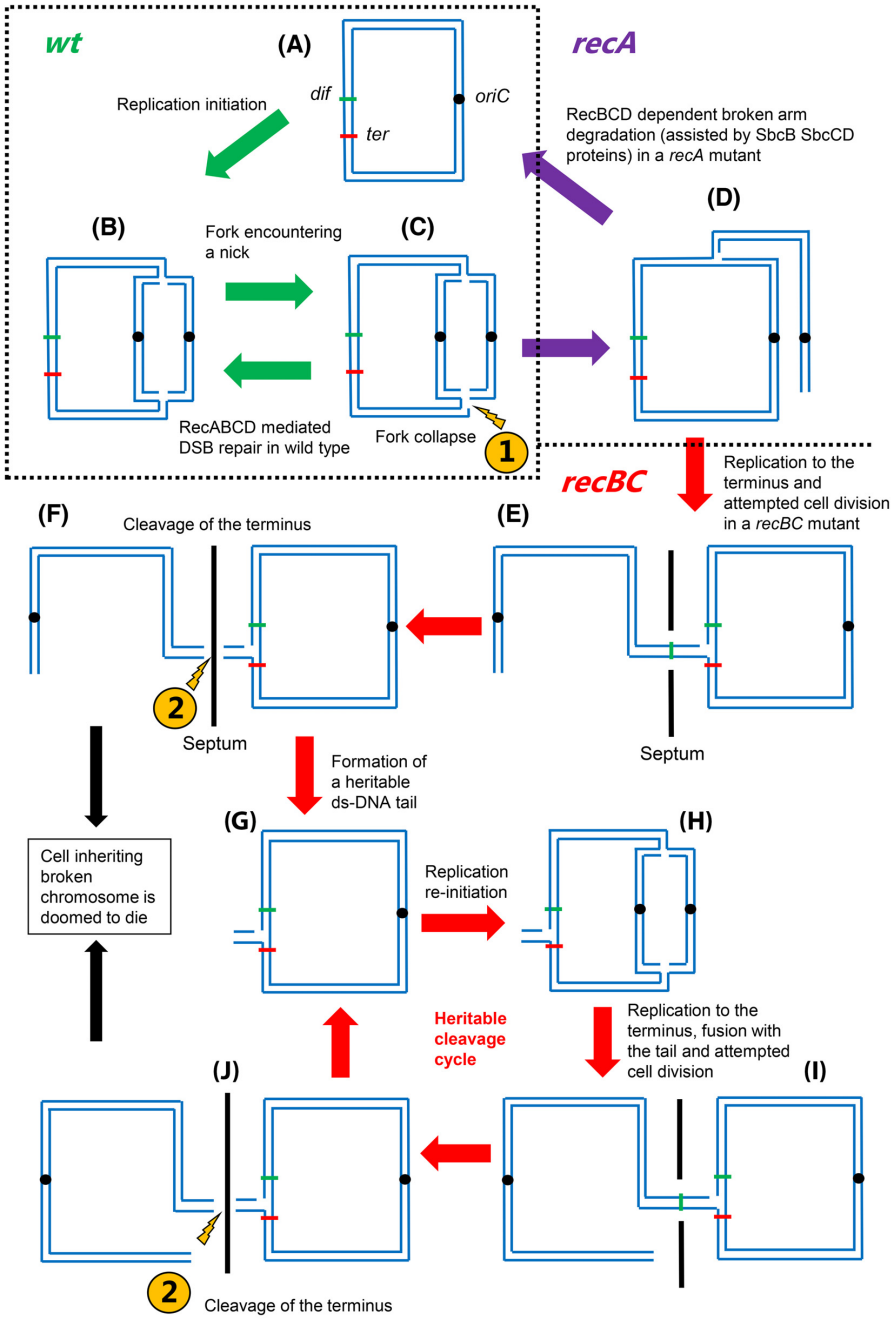
Processing of a broken replication fork in wild type (*wt*), Δ*recA* and Δ*recBCD* cells. DNA replication initiates at a fixed position known as *oriC* (denoted by a small circle) on a circular chromosome (**a**) and progresses bidirectionally (**b**). Encounter of the replication fork with a nick on the template strand (**c**) leading to a broken replication fork (replication fork collapse) and generation of a DSE (one-ended DSB) at the fork (depicted by 1 in the figure). This can lead to cell division-dependent DNA cleavage of the terminus in *recBC* mutant cells (depicted by 2 in the figure). **In *wt* cells**, the broken fork is repaired by RecBCD-RecA mediated recombinational repair, to re-establish an intact replication fork (from **c** to **b**). **In Δ*recA* cells**, RecBCD binds to this DSE and degrades the entire broken arm, to generate an intact single circular chromosome (from **d** to **a**). However, the degradation process of the entire broken arm by RecBCD needs assistance from exonucleases (such as ExoI, SbcCD etc.), since frequent encounter with Chi sequences by RecBCD leads to the generation of long 3′-ssDNA overhangs and disassociation of RecBCD. Owing to its ability to bind only blunt (or nearly blunt) DSEs, RecBCD is unable to bind and restart degradation from these long 3′ ssDNA overhangs. ssDNA exonucleases degrade the overhangs to make them blunt and so facilitate RecBCD loading and further degradation. **In Δ*recBCD* cells**, the broken arm persists. The other intact replication fork continues to progress towards the terminus (e) and reaches an appropriately oriented *ter* site (shown here as red line). Since replication and segregation occur simultaneously in bacterial cells, the intact circular chromosome and the broken linear chromosome segregate into two halves of the cell but remain linked by the intact fork in the terminus (**e**). FtsK action aligns two *dif* sites (shown here as green lines), one on the circular and another on the linear chromosome, in the middle of the septum. Septum formation causes guillotining of the chromosome in the *dif* region of the terminus, separating linear and circular chromosomes into two daughter cells (**f**). The cell inheriting the linear chromosome does not grow further. The cell inheriting the circular chromosome has a replication fork attached to a short linear DNA tail that remains in the terminus (**g**). A new round of replication initiated at *oriC* sends forks around the chromosome (**h**), which merge with the previous fork in the terminus, causing the short linear tail to be enlarged to an entire broken chromosome arm (**i**). Attempted segregation followed by guillotining (**j**) makes the process heritable (from **g** to **j** and then back to **g**).

This dual realization that the location of DNA loss observed in a *recBC* mutant did not correspond to the location of replication termination and that the terminus breaks are only generated in the *recBC* mutant led to the proposal of a second model (Sinha *et al*. 2018). In this second model (Fig. 4), randomly located breakage of a DNA replication fork leads to the formation of a sigma-replicating chromosome. The broken fork cannot be repaired in a *recBC* mutant, so the sigma-replicating chromosome persists (Fig. 4cde). This then causes a problem when the cell tries to segregate its replicated chromosomes and divide (Fig. 4e). The terminus region of one of the chromosomes (the linear tail of the sigma structure) becomes trapped as the septum attempts to close and this attempted cell division leads to chromosome breakage (Fig. 4f). The initial break of the replication fork is randomly located on the chromosome and so cannot be detected in a population of cells (Fig. 4c). It is independent of *recBC* but persists in a *recBC* mutant. The subsequent break occurs in the *dif*/*terC* region but only if the cell is a *recBC* mutant, because only in the absence of replication fork repair by RecBCD does the sigma-replicating chromosome persist and get trapped in the closing septum at cell division (Fig. 4e-f). This subsequent break in the *dif*/*terC* region generates one cell with a linear chromosome, which is doomed to die, and a second cell with a circular chromosome containing a short linear tail in the terminus (Fig. 4g). If this mark persists until the arrival of the next round of replication forks, a new sigma-shaped chromosome is generated and the cleavage cycle is repeated causing the heritability of terminus breakage that has been documented (Fig. 4g-j) (Sinha *et al*. 2017, 2018).

This model has been confirmed by many tests. MFA by genomic sequencing has shown that terminus DNA loss in a *recBC* mutant is not dependent on the action of *dif*/XerCD but is dependent on cell division and is focused in the *dif/terC* region by the action of FtsK (Sinha *et al*. 2017). Fluorescence microscopy, in which yGFP-ParB_pMT1_ binds to a parS_pMT1_ site inserted between *dif* and *terC*, has been used to analyze terminus DNA loss in the *recBC* mutant at a single cell level (Sinha *et al*. 2017, 2018). In *recBC* mutant cells, the terminus is normally replicated and two foci are clearly visible after replication. In 18% of cell divisions, one of these two foci is lost and one daughter cell is born without a focus and does not grow further. The other cell, which keeps a focus, grows and again replicates the parS_pMT1_ site so that two foci become visible. However, one focus is again lost during division making this DNA loss heritable. The cell with the linear chromosome that loses its focus does not grow because of the action of the HipA toxin, which is more stable than the HipB antitoxin encoded by the *hipBA* operon that is located in the terminus and is degraded rapidly following DSB formation (Sinha *et al*. 2018). More than two terminus foci were never observed, despite cell division-mediated focus-loss being observed much later than replication was complete, contrary to a prediction of amplification preceding DNA loss, as proposed in the first model. After the cell division induced breakage of the chromosomes in the *recB* mutant, the DSEs generated at the terminus are degraded by exonucleases and SbcCD plays major role in that end degradation (Dimude, Midgley-Smith and Rudolph 2018). Further evidence that collisions of replication forks are not implicated in terminus DNA loss was obtained by investigating the phenomenon in a phage N15 lysogen where the TelN gene product processes a *tos* site to generate a linear chromosome with two hairpin capped ends. In cells with this linear chromosome, *recBC* dependent terminus DNA loss was still observed despite converging replication forks not meeting in the terminus (Dimude, Midgley-Smith and Rudolph 2018; Sinha *et al*. 2018). The pattern of degradation was affected by the presence or absence of the HipA HipB toxin-antitoxin system with the cell inheriting a broken chromosome containing the *hipBA* operon being poisoned by the HipA toxin and showing little degradation (Sinha *et al*. 2017; Dimude, Midgley-Smith and Rudolph 2018). Moving the site of linearization 200 kb away from the *hipBA* operon and *dif*/terC allowed more symmetrical degradation and a displaced location of maximum degradation, consistent with a delay to the degradation of *hipBA* and a preferred location of cleavage that is determined by the DNA lying in the path of the septum when the cell tries to divide, rather than the presence of a specific site of cleavage (Dimude, Midgley-Smith and Rudolph 2018).

### Conclusions from the terminus degradation studies

Studies of terminus DNA loss observed in a *recBC* mutant have revealed a new and unexpected phenomenon, the cleavage of a sigma-replicating chromosome at cell division (Fig. 4e-j) (Sinha *et al*. 2018). The mechanism of this cleavage is still unknown and may or may not be related to the guillotining of chromosome dimers in *dif* or *xerCD* mutants (Hendricks *et al*. 2000; Prikryl, Hendricks and Kuempel 2001). The work by Sinha et al (2018) has also provided an estimate for the frequency of replication fork breakage of 18% of replication cycles in cells growing in a minimal glucose medium at 30 °C. The heritability of the phenomenon explains the overall poor viability of the *recBC* mutant. The tests of the mechanism show that the loss of DNA in the terminus occurs because of breaks that occur during cell division only in *recBC* mutant cells, but the initiating event is the formation of a sigma-replicating chromosome by lack of repair of a broken DNA replication fork. In the *recBC* mutants, the terminus DNA ends, generated by cleavage, are degraded by other nucleases, with an important role for SbcCD (Fig.   4f) (Dimude, Midgley-Smith and Rudolph 2018).

## GENERAL CONCLUSIONS

Several studies in the last decade have provided vital novel insights and have given different perspectives on the roles of DNA replication and DSB repair proteins in maintaining bacterial chromosome integrity and stable DNA replication (SDR). Thanks primarily to studies of the bacterium *E. coli*, now we know that SDR induced in many of these mutants (not only in *rnhA*, where it was originally characterized) has the potential to replicate the entire chromosome and to permit survival without *oriC* mediated replication initiation. Remarkably, chromosome replication by SDR has only two obstacles to overcome: 1. to cross fork trap barriers and, 2. to survive the consequences of head-on conflicts with the transcriptional complex. Amazingly, *E. coli* chromosome organization and replication-transcription orientation have evolved in a way that efficiently uses these two restrictions to ensure that any chromosomal region except the terminus is replicated in only one direction. Recombination-dependent, origin-independent DNA replication has not yet been observed for other bacterial species. However, our increased understanding of the *E. coli* system opens up the possibility of making such observations using appropriate mutants. It is a well-known mechanism of DNA replication in the late lytic cycle of bacteriophage T4 and could permit the survival of the archaeon *H. volcanii* lacking its four origins of DNA replication. It may indeed represent a primitive mechanism of DNA replication initiation.

*E. coli* terminus DNA amplification has been proposed to be associated with aberrant events, hypothesized to occur when DNA replication forks collide, in the absence of key proteins that normally remove hypothetical aberrant DNA structures. However, we question whether the merging of DNA replication forks actually leads to such aberrant events in living cells. Further studies are required to clarify the mechanisms responsible for terminus DNA amplification in *E. coli* and to determine whether similar events can be observed in other bacterial species. Terminus DNA loss in an *E. coli recBC* mutant has revealed the existence of a new mechanism for the generation of DSBs, which involves the cleavage of DNA trapped at the septum during cell division. The details of this cleavage mechanism are yet to be understood, its phylogenetic distribution revealed, and its evolutionary value determined.
